# Accuracy and Safety of the 15-Day CareSens Air Continuous Glucose Monitoring System

**DOI:** 10.1089/dia.2023.0468

**Published:** 2024-03-28

**Authors:** Kyung-Soo Kim, Seung-Hwan Lee, Won Sang Yoo, Cheol-Young Park

**Affiliations:** ^1^ Department of Internal Medicine, CHA Bundang Medical Center, CHA University School of Medicine, Seongnam, Korea.; ^2^ Division of Endocrinology and Metabolism, Department of Internal Medicine, Seoul St. Mary's Hospital, College of Medicine, The Catholic University of Korea, Seoul, Korea.; ^3^ Division of Endocrinology and Metabolism, Department of Internal Medicine, Dankook University College of Medicine, Cheonan, Korea.; ^4^ Department of Internal Medicine, Samsung Kangbuk Hospital, Sungkyunkwan University School of Medicine, Seoul, Korea.

**Keywords:** Blood glucose, Diabetes mellitus, Glucose, Safety, Technology

## Abstract

**Background:**

We evaluated the accuracy and safety of the CareSens Air, a novel real-time continuous glucose monitoring system (CGMS), during 15 days of use in adults with diabetes.

**Methods:**

Adults with either type 1 diabetes or type 2 diabetes requiring intensive insulin therapy participated at four sites in South Korea. All participants wore the sensor for 15 days. Participants were scheduled for four 8-h clinic sessions on Day 1, 5 ± 1, 10 ± 1, and 15. Accuracy was evaluated based on the proportion of continuous glucose monitoring (CGM) values within 15% of YSI values ≥100 mg/dL or within 15 mg/dL of YSI values <100 mg/dL (%15/15), along with the %20/20, %30/30, and %40/40 agreement rates. The mean absolute relative difference (MARD) between the CGM and YSI values was calculated.

**Results:**

Data from 83 participants (83 sensors, 10,029 CGM-YSI matched pairs) were analyzed. The overall MARD was 10.42%, and the overall %15/15, %20/20, %30/30, and %40/40 accuracy were 78.55%, 89.04%, 96.47%, and 98.87%, respectively. The consensus error grid analysis showed that 99.92% of CGM values fell into Zone A or B (Zone A: 89.83%, Zone B: 10.09%). The %20/20 accuracy of CGMS was 88.11% on Day 1, 90.11% on Day 3–5, 92.09% on Day 8–10, and 85.63% on Day 15. No serious adverse events were reported.

**Conclusions:**

The CareSens Air demonstrated accurate performance across the wide glycemic range and was well tolerated during the 15-day sensor use period.

## Introduction

Continuous glucose monitoring systems (CGMSs) have given patients the ability to use real-time glucose data to help them achieve tighter glycemic control.^1–5^ Patients with type 1 and type 2 diabetes, and especially those who require insulin therapy, increasingly use CGMs to improve and maintain glycemic control.^6–8^ The American Diabetes Association recommends that real-time CGM or intermittently scanned CGM be offered for management of adults with diabetes on multiple daily injections or continuous subcutaneous insulin infusions who can use the devices safely by themselves or with a caregiver.^9^

However, the first-generation CGMS had low accuracy, with an estimated mean absolute relative difference (MARD) of 12.8%–16.7%.^10,11^ Further, since the first-generation CGMS only worked for three to seven consecutive days, its applicability and use were limited. In the past two decades, the CGMS has improved in accuracy and now has an extended wearing time up to 14 days.^12–14^ In addition, other features such as patient comfort improved with technological improvements. Nevertheless, there is a continued research effort to develop new CGM sensors that meet the ideal technological requirements such as sensor size, lifetime, and capabilities.

The CareSens Air is the first CGM device developed by the South Korean medical device company i-sens, Inc., (Incheon, South Korea) that has been approved by the South Korean Ministry of Food and Drug Safety as an adjunctive use device. It is the fourth CGM device to be approved in South Korea, following approvals for the pre-existing products made by Medtronic, Abbott, and Dexcom. The CareSens Air is the smallest and lightest CGM device available in South Korea. It can be used for 15 consecutive days and features a calibration mechanism for reliability. In this study, we evaluated the accuracy and safety of the CareSens Air during 15 days of use in adult patients with diabetes.

## Methods

### Study design and participants

This prospective, multicenter, single-arm, open-label, pivotal study was conducted between February and August 2022 at four sites in South Korea. Before initiating the study, approval was obtained from the Institutional Review Board of each participating institution regarding the clinical trial protocol, subject information sheet and consent form, materials and procedures related to subject recruitment, and other relevant documents. This study was conducted, evaluated, and recorded in accordance with the Korean medical device clinical trial management standards, the International Conference on Harmonization-Good Clinical Practice, and the Helsinki Declaration. It also abided by domestic laws and relevant regulations. Written informed consent was obtained from each study participant before study initiation.

We enrolled patients aged 19 years or older with type 1 diabetes, or type 2 diabetes requiring intensive insulin therapy. All patients participated in a clinic session involving venous blood draws. The exclusion criteria were skin disorder at the insertion site, allergy to medical adhesives, history of severe hypoglycemia, cerebrovascular or cardiovascular diseases, epilepsy, syncope, adrenal diseases within 6 months, hematocrit <15% or >65%, hemoglobin <13 g/dL in men or <12 g/dL in women, renal diseases requiring dialysis, prescribed medication that could affect glucose metabolism (e.g., glucocorticoid) or YSI analyzer (e.g., methimazole, isoniazid, resorcinol, P-phenylenediamine), pregnancy, and refusal to provide written consent.

A total of 89 participants underwent eligibility assessment. Five patients were excluded because of screening failure (*n* = 4) and severe acute respiratory syndrome coronavirus 2 infection (*n* = 1). Finally, 84 participants were included.

### Study procedure

The participants wore two sensors (one on the back of each upper arm) for 15 days. The dimensions of the wearable transmitter are 35.2 × 19.2 × 5.0 mm (Supplementary Fig. S1). Over-patch tape was provided, and participants were allowed to use them as needed. Participants could access the CGM data via the user application, but they were blinded to the CGM data only during the clinic sessions. Sensor insertions were performed in the clinic by the research team. After a stabilization period of 2 h following insertion of the sensor, the CGMS calibration was performed using self-monitoring blood glucose (SMBG) (CareSens Dual, i-SENS, Inc., Wonju, South Korea). During the sensor use period, participants underwent CGMS calibration as instructed. On the first day, user calibration was conducted at two instances, separated by a 12-h interval. After the first day, calibration was carried out once every 24 h.

At the screening visit, we measured glycated hemoglobin (HbA1c) using a high-performance liquid chromatography. The participants were scheduled for four 8-h clinic sessions on Day 1, 5 ± 1, 10 ± 1, and 15. Participants were not required to fast overnight. They ate meals without carbohydrate restriction at scheduled times and made their usual insulin dosing decisions. During these sessions, venous blood samples were collected through an intravenous catheter placed in the arm at ∼15 ± 5 min intervals. Glucose concentrations were measured using the YSI 2300 STAT PLUS Glucose and Lactate Analyzer (YSI, Inc., Yellow Springs, OH). All YSI data were included for analysis even when the blood sample was hemolyzed. No insulin or meal challenge tests were performed during the clinic session. Following the POCT-5A guidelines, SMBG measurements at 6-h intervals within the 24-h period were performed for the calibration stability test on either Day 5–6 or Day 10–11.^15^

All CGM removals were performed by the research team. During the removal visit on Day 16, the research team evaluated insertion sites and adhesive areas, and documented any participants-reported adverse events. The participants were asked to rate their satisfaction with the CGM sensor on a 7-point Likert scale. On the seventh day after the CGMS sensor was removed, participants were contacted via telephone to monitor for any adverse events.

To ensure consistent glucose measurements across clinical sites, the harmonization method involves standardizing the calibration process of the YSI analyzers. This is achieved by using predetermined quality control materials such as JCCRM521 standards and YSI Glucose/Lactate Standards at each site. Operators are required to perform daily calibrations before testing participant samples, ensuring that readings fall within an acceptable range. In addition, a two-point calibration is conducted immediately before and after a set of blood sample measurements, with all results meticulously recorded.

### Data analysis

CGM data were saved in a database file within the user application. The data were anonymized and analyzed after the study was completed. YSI measurements performed during the clinic sessions were matched with the first available CGM value obtained within the subsequent 5 min from a primary sensor attached to a participant. The primary sensor was randomly selected from two available sensors for a participant just before being attached. Only matched data pairs in which the sensor results were within the reportable range (40–500 mg/dL) were used for the performance evaluation. To analyze accuracy in terms of %15/15, %20/20, %30/30, and %40/40, the percentage of CGM measurements that met the criteria of ±15%, ±20%, ±30%, or ±40% was compared with YSI values (when YSI values were ≥100 mg/dL) or ±15, ±20, ±30, or ±40 mg/dL (when YSI values were <100 mg/dL) was calculated. The MARD was calculated as the average of the absolute relative differences between the CGM and YSI values. The mean absolute difference (MAD) was calculated as the average of the absolute differences between CGM and YSI values. Clinical accuracy was determined using consensus error grid (CEG) analysis.^16^ Rate of change (RoC) was calculated as the difference between consecutive glucose values per unit of time, in units of mg/dL per minute. MARD and %20/20 were evaluated for the following RoC categories: less than −3, −3 to less than −2, −2 to less than −1, −1 to 1, >1 to 2, >2 to 3, and >3.

To evaluate the CGM sensor stability, the paired CGM-YSI values were grouped into four periods: (1) beginning (Day 1); (2) early middle (Day 3–5); (3) late middle (Day 8–10); and (4) end (Day 15). Paired absolute relative difference (PARD) was calculated based on the paired data from two sensors attached to a participant. The lag time was determined using a regression method that identifies the optimal time shift between CGM data and reference data by seeking the time shift that yields the highest coefficient of determination from the regression analysis.^17^

The true alert rate was determined by the proportion of times the CGMS readings would trigger alerts for low or high blood glucose based on the retrospective analysis, and YSI values were either below the threshold for hypoglycemia (54 or 70 mg/dL) or above the threshold for hyperglycemia (250 mg/dL) within 15 and 30 min before and after the alert. The detection rate was retrospectively examined, which is the proportion of times the CGMS would trigger alerts within 15 and 30 min when the YSI readings were below or above the set thresholds for hypoglycemia or hyperglycemia, respectively.

Safety was assessed by monitoring adverse events during the sensor use period. The final evaluation analysis was performed using all the available data from participants. All statistical analyses were performed using SAS Software (Version 9.4).

## Results

The baseline characteristics of the participants are presented in Table 1. The mean age was 40.10 years, and the mean body mass index was 24.60 kg/m^2^. Among 84 participants, 54.76% were female, 75% had type 1 diabetes, and the mean duration of diabetes was 15.57 years. Most participants used multiple daily injections (92.86%). The mean HbA1c level was 7.82%. Among 84 participants, 1 participant did not qualify for the clinic sessions due to accidental detachment of both sensors before the first clinic session. The remaining 83 participants provided data from 83 sensors (10,051 CGM-YSI matched pairs). Of these matched pairs, there were 10,029 in the reportable range of 40–500 mg/dL that were included in the calculation of accuracy metrics. The mean glucose level measured by YSI was 165 mg/dL (Supplementary Fig. S2). The percentage of YSI readings that was below 80 mg/dL was about 4.6%, and the percentage higher than 300 mg/dL was 4.4%.

**Table 1.** Baseline Characteristics of Participants (*N* = 84)

Demographics	Value
Age (years)	
Mean (SD)	40.10 (14.54)
Median	38.00
Min, Max	19.00, 77.00
Sex	
Male	38 (45.24)
Female	46 (54.76)
Height (cm)	
Mean (SD)	165.70 (8.95)
Median	165.05
Min, Max	146.60, 186.00
Weight (kg)	
Mean (SD)	68.07 (15.57)
Median	63.00
Min, Max	40.60, 118.50
Body mass index (kg/m^2^)	
Mean (SD)	24.60 (3.99)
Median	23.40
Min, Max	15.90, 37.00
Duration of diabetes (years)	
Mean (SD)	15.57 (9.50)
Median	13.91
Min, Max	0.81, 42.54
Type of diabetes mellitus	
Type 1 diabetes mellitus	63 (75.00)
Type 2 diabetes mellitus	21 (25.00)
Mode of insulin injection	
Multiple daily injections	78 (92.86)
Continuous subcutaneous insulin infusion	6 (7.14)
Glycated hemoglobin (%)	
Mean (SD)	7.82 (1.94)
Median	7.50
Min, Max	5.00, 17.10

Data are presented as mean ± standard deviation or number (%).

SD, standard deviation.

**FIG. 1.**
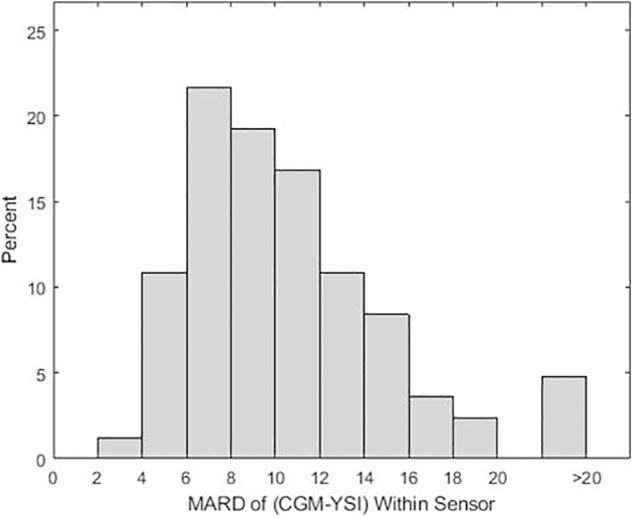
Aggregated sensor MARD (%) of CGM-YSI histogram plot. CGM, continuous glucose monitoring; MARD, mean absolute relative difference.

The overall MARD was 10.42%, and the overall %15/15, %20/20, %30/30, and %40/40 accuracy were 78.55%, 89.04%, 96.47%, and 98.87%, respectively (Table 2). The overall MAD was 16.73 mg/dL. Among 83 sensors, 65 (78.3%) had >80% of CGM-YSI matched pairs that met the %20/20 accuracy criterion (Fig. 1).

**Table 2.** Sensor Accuracy Across Various Glucose Ranges

Glucose range, mg/dL	Matched pairs (*n*)	Percentage within 15%/15 mg/dL	Percentage within 20%/20 mg/dL	Percentage within 30%/30 mg/dL	Percentage within 40%/40 mg/dL	MAD (mg/dL)	MARD (%)
Overall	10,029	78.55%	89.04%	96.47%	98.87%	16.73	10.42
<54	17	76.47%	88.24%	100%	100%	11.88	NA
54–69	152	67.11%	77.63%	96.05%	98.68%	12.38	NA
70–180	6345	78.41%	88.37%	96.19%	99.04%	NA	10.53
181–250	2346	81.54%	92.63%	97.31%	98.51%	NA	9.40
>250	1169	74.85%	87.00%	96.32%	98.72%	NA	10.42

MAD, mean absolute difference; MARD, mean absolute relative difference.

Accuracy across days of sensor use is presented in Table 3. At all-time points, the MARD was <12% and the MAD was <19 mg/dL. The %20/20 accuracy of CGMS compared with YSI measurements was 88.11% on Day 1, 90.11% on Day 3–5, 92.09% on Day 8–10, and 85.63% on Day 15. The %40/40 accuracy was >98% at all time points. Accuracy by diabetes type is presented in Table 4. The MARD was 10.62% in patients with type 1 diabetes and 9.85% in patients with type 2 diabetes.

**Table 3.** Sensor Accuracy Across Days of Sensor Use

Time from insertion (days)	Matched pairs (*n*)	Percentage within 15%/15 mg/dL	Percentage within 20%/20 mg/dL	Percentage within 30%/30 mg/dL	Percentage within 40%/40 mg/dL	MAD (mg/dL)	MARD (%)
Day 1	2633	78.12%	88.11%	96.28%	98.59%	16.97	10.70%
Days 3–5	2589	81.77%	90.11%	95.94%	98.88%	15.61	9.70%
Days 8–10	2489	82.16%	92.09%	98.35%	99.60%	16.10	9.63%
Day 15	2318	71.57%	85.63%	95.25%	98.40%	18.40	11.74%

**Table 4.** Sensor Accuracy by Diabetes Type

Diabetes type	No. of subjects	Matched pairs (*n*)	Percentage within 15%/15 mg/dL	Percentage within 20%/20 mg/dL	Percentage within 30%/30 mg/dL	Percentage within 40%/40 mg/dL	MARD (%)
Type 1	62	7374	77.41%	88.43%	96.42%	99.04%	10.6*2*
Type 2	21	2655	81.73%	90.73%	96.61%	98.42%	9.85

**FIG. 2.**
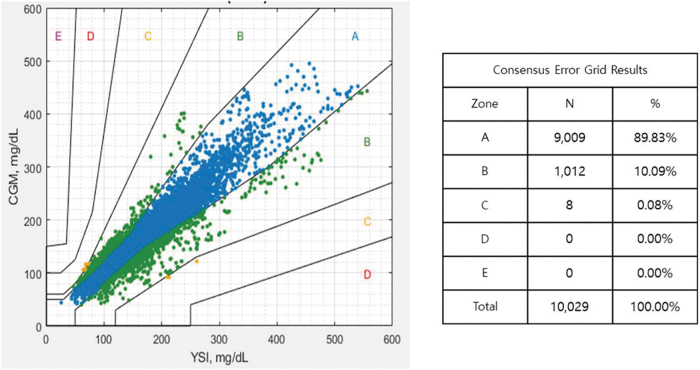
Consensus error grid analysis plot for CGM (CSAir) versus YSI values. CSAir, CareSens Air.

To compare the sensor measurements with YSI values, scatter plots were used to display the CGM measurements in relation to YSI values on the CEG (Fig. 2). Based on analysis of the CEG, the percentage of CGM values in Zones A and B was 99.92% (Zone A: 89.83%, Zone B: 10.09%). The percentage of CGM values in Zone C, which could potentially influence the clinical outcomes, was 0.08%. There were no measurements in Zone D and E, which could cause significant medical risks or hazardous outcomes.

To evaluate the stability of daily calibration, the accuracy of the CGMS was measured against SMBG values at 6-hourly intervals over a 24-h period (Supplementary Table S1). The %20/20 accuracy measurements of the CGMS were as follows on Day 5–6: 0–6 h: 81.90%; 6–12 h: 81.62%; 12–18 h: 92.92%; and 18–24 h: 91.85%. On Day 10–11, the %20/20 accuracy measurements were 85.41% at 0–6 h, 91.19% at 6–12 h, 91.89% at 12–18 h, and 93.85% at 18–24 h.

Concurrence matrix for seven RoC categories is presented in Table 5. Mean absolute RoC was 0.73 mg/(dL·min). The agreement between sensor and reference was 73.67%. No five or six categories difference was observed. The highest %20/20 agreement rates and lowest MARDs occurred when CGM readings were increasing or decreasing by no more than 1 mg/dL per minute (Supplementary Table S2). The MARDs at rapidly decreasing (RoC less than −3 mg/dL per minute) or rapidly increasing (RoC >3 mg/dL per minute) were 16.30% and 12.09%, respectively, and the %20/20 agreement rates were 67.77% and 81.91%, respectively.

**Table 5.** Concurrence Matrix for Seven Rate of Change Categories

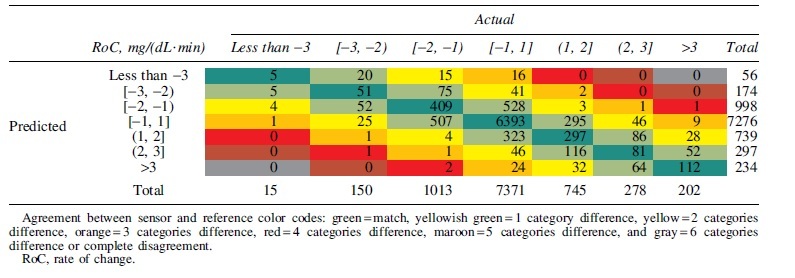

Table 6 summarizes sensor alert and detection rates for hypoglycemia and hyperglycemia at various thresholds. When the CGMS alerted for hypoglycemia, the proportion of YSI readings below 54 mg/dL was 11.9%, and below 70 mg/dL was 55.1% within 15 min of the alarm. These rates were 11.9% and 56.3% within 30 min of the alarm, respectively. For hyperglycemia alarms, the YSI readings above 250 mg/dL were 88.3% within 15 min and 91.4% within 30 min. When YSI readings were below 54 mg/dL, the CGMS alarmed for hypoglycemia 41.2% of the time within 15 min and 47.1% within 30 min. For readings below 70 mg/dL, these rates were 65.7% and 71.6%, respectively. When YSI readings were above 250 mg/dL, the CGMS hyperglycemia alarm sounded 80.9% of the time within 15 min and 84.6% within 30 min.

**Table 6.** Hypoglycemic and Hyperglycemic Alerts and Detections at Various Thresholds

Condition	Time (minutes)	Alert or detection level (mg/dL)	Total number of alerts	Number of true alerts	Total number of detections	Number of true detections
Hypoglycemia	Within ±15 min	54	42	5 (11.9%)	17	7 (41.2%)
		70	167	92 (55.1%)	169	111 (65.7%)
	Within ±30 min	54	42	5 (11.9%)	17	8 (47.1%)
		70	167	94 (56.3%)	169	121 (71.6%)
Hyperglycemia	Within ±15 min	250	1023	903 (88.3%)	1171	947 (80.9%)
	Within ±30 min	250	1023	935 (91.4%)	1171	991 (84.6%)

Data are presented as number (%).

The PARD was 8.14%. The overall mean lag time between the sensor and YSI measurements was 5.2 ± 3.7 min. There were no serious adverse events reported during the study. Five participants reported mild adverse events, as follows: one experienced mild contact dermatitis, one experienced mild dyspepsia, one experienced mild pyrexia, one experienced mild cystitis, and one experienced a foot fracture that was unrelated to the CGM sensor.

The mean scores on the 7-point Likert scale for the statements “attaching the CGMS sensor is less painful than finger pricking” and “the method of wearing the CGMS is convenient” were 6.63 and 6.23, respectively (Supplementary Table S3).

## Discussion

The CareSens Air CGM produced accurate glucose readings with MARD 10.42% in adult patients with diabetes. In clinic sessions, accurate readings from the CGM sensor were confirmed by frequent blood glucose samplings in a broad range of glucose levels. Further, 99.92% of CGM values fell into Zones A and B, meaning that they either have no impact on clinical decision making or have minimal to no impact on clinical outcomes. The sensor performance was maintained throughout the 15-day wear duration. There were no safety concerns during the study.

In a study of pregnant women with diabetes using the G6 system without intentional glucose manipulations, the overall MARD and that of sensors worn on the abdomen, upper buttock, and posterior upper arm was 10.3%, 11.5%, 11.2%, and 8.7%.^18^ In a study of the G7 system (Dexcom), the overall MARD of arm-placed sensors was 8.2% and that of abdomen-placed sensors was 9.1%, when hyperglycemia and hypoglycemia were induced by adjusting the timing or amount of rapid-acting insulin and carbohydrates.^13^ When glucose levels were manipulated to induce high or low blood glucose levels through carbohydrate consumption and insulin timing during the clinic sessions, the overall MARD of the FreeStyle Libre 2 system was 9.2% for adult participants and 9.7% for pediatric participants.^19^ The overall MARD of the FreeStyle Libre 3 system against the YSI reference was 7.8% in the study without insulin or meal challenges.^14^ Although it is difficult to directly compare because the study procedures including intentional glucose manipulation and data characteristics are different, the overall MARD of the CareSens Air CGM sensors (10.42%) seems to be similar to or slightly higher than those of other CGMSs.

CareSens Air has several advantages. It is a real-time CGM that can be used for 15 days. Users can know their glucose values without any scanning. And it has alert and alarm options. In addition, CareSens Air is an integrated sensor-transmitter. It is pre-assembled within the applicator, and users can attach it by simply pressing the release button after removing the safety cap. Because it has a slender profile of just 19.2 mm wide, the sensor will be suitable for attachment to the back of the upper arm. However, the CareSens Air requires calibration once every 24 h, unlike the FreeStyle Libre or the Dexcom G7.

Some limitations should be considered when interpreting the results of this study. First, although many studies with other CGMs performed hyperglycemic or hypoglycemic challenge tests, this study did not. We were able to evaluate the accuracy of the CareSens Air under conditions of natural glycemic variation, but there were a limited number of hypoglycemic or hyperglycemic events. Nevertheless, we should consider that CareSens Air was approved as an assistive use device. Second, the study population had relatively good glycemic control (mean HbA1c 7.82%). This is another reason why the number of hypoglycemic or hyperglycemic events is limited. Third, we could not get the information for patients treated with less intensive insulin therapy (such as basal insulin alone) with oral antidiabetic drugs (OADs) or those on OADs alone. Finally, this study excluded children and adolescents. Further evaluation in this population is needed.

In conclusion, the CareSens Air CGM is accurate and safe for up to 15 days of use in adult patients with diabetes.

## Supplementary Material

Supplemental data
