# Supplementary material for: Accuracy and Safety of the 15-Day CareSens Air Continuous Glucose Monitoring System
Source: Diabetes Technol Ther. 2024 Mar 28;26(4):222–8. doi: 10.1089/dia.2023.0468 (PMC10979678; doi:10.1089/dia.2023.0468)
Supplement: Supplemental data [file Suppl_Figures_Tables.pdf]

Supplementary Figure Legends

Supplementary Figure 1. The wearable sensor/transmitter and the applicator of the CareSens Air. The wearable sensor/transmitter measures 35.2 x 19.2 x 5.0 mm.

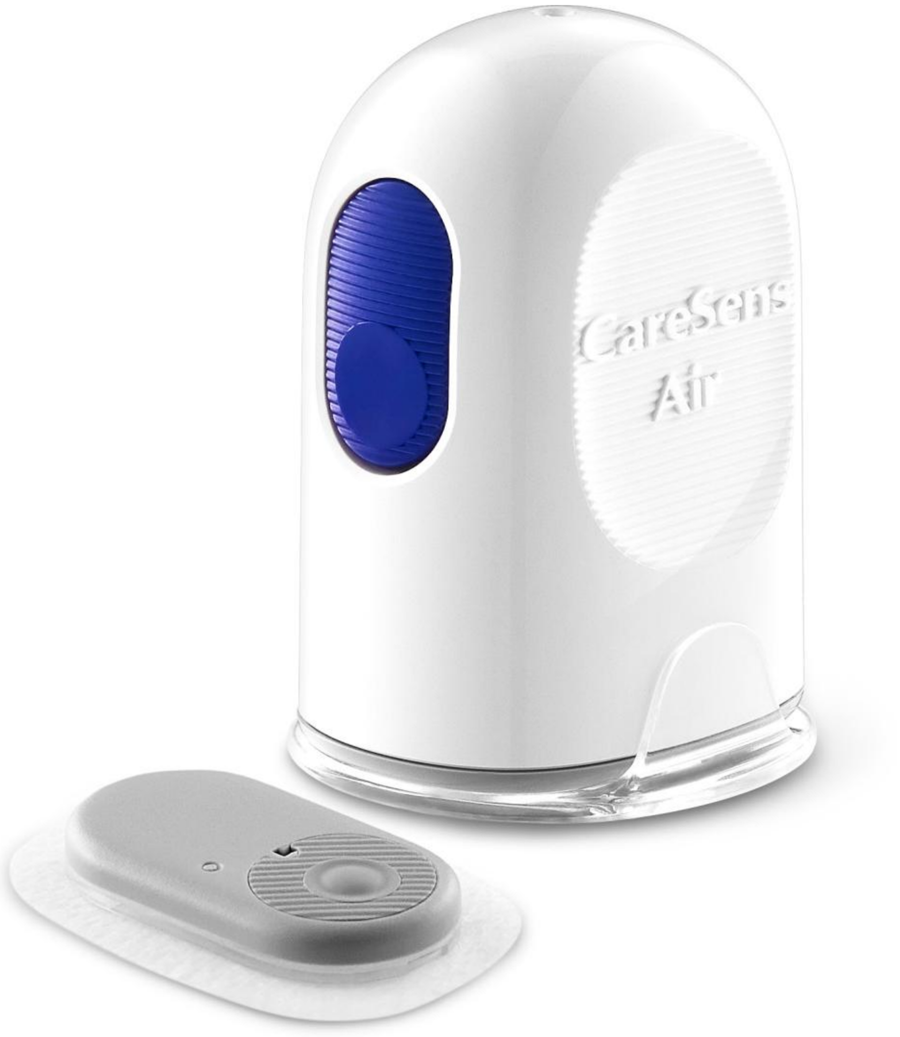

Supplementary Figure 2. Distribution of blood glucose levels measured by YSI.

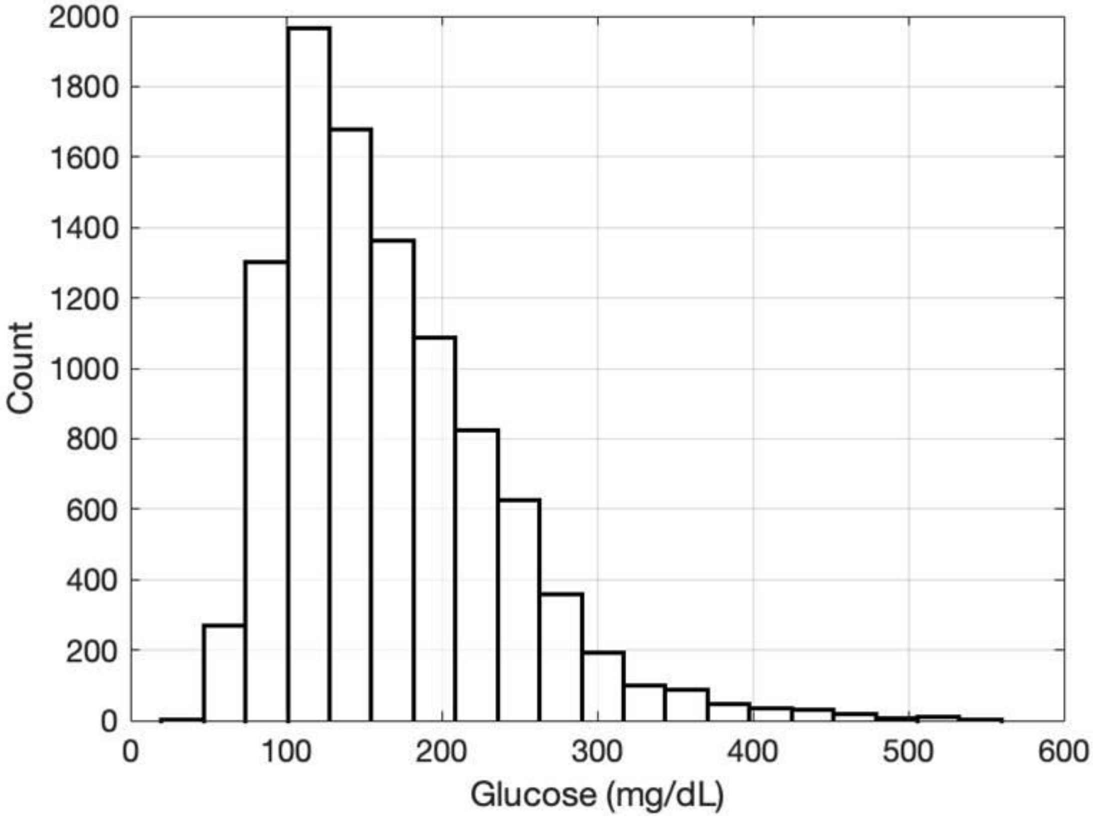

Supplementary Table 1. Sensor accuracy compared to self-monitoring blood glucose values

Time from calibration (hours)		Matched pairs (n)	Percentage within 15%/15 mg/dL	Percentage within 20%/20 mg/dL	Percentage within 30%/30 mg/dL	Percentage within 40%/40 mg/dL
Days 5-6	0 – 6	536	70.15%	81.90%	92.72%	96.64%
	6 – 12	272	68.38%	81.62%	88.97%	94.12%
	12 – 18	1,214	83.36%	92.92%	98.11%	99.01%
	18 – 24	1,154	80.07%	91.85%	98.79%	99.74%
Days 10-11	0 – 6	370	78.92%	85.41%	94.32%	99.46%
	6 – 12	159	83.02%	91.19%	95.60%	99.37%
	12 – 18	1,184	83.19%	91.89%	98.06%	99.32%
	18 – 24	1,187	85.59%	93.85%	98.99%	100.00%

Supplementary Table 2. Sensor accuracy at various rate of glucose concentration change

CGM rate range (mg/dL per minute)	Matched pairs (n)	Percentage within 20%/20 mg/dL	MARD (%)
<-3	121	67.77%	16.30
-3 to <-2	253	83.00%	12.26
-2 to <-1	997	88.67%	11.09
-1 to 1	7,219	90.83%	9.95
>1 to 2	809	86.28%	10.54
>2 to 3	348	77.01%	13.12
>3	282	81.91%	12.09

CGM, continuous glucose monitoring; MARD, mean absolute relative difference.

Supplementary Table 3. The scores of satisfaction questionnaire

No.	Question	Score
1	The CGMS is more convenient for glucose management than fingerstick glucometers.	6.40 (1.04)
2	Attaching the CGMS sensor is less painful than finger pricking.	6.63 (0.59)
3	The method of wearing the CGMS is convenient.	6.23 (1.09)
4	The size, shape, and weight of the CGMS sensor are suitable for wear.	6.10 (1.24)
5	Education on how to wear and use the CGMS was helpful.	6.44 (0.78)
6	Problems can be solved by following the instructions in the user manual.	5.69 (1.27)
7	When assistance is needed for using the CGMS, referring to the user manual is preferred over calling the help center.	4.89(1.63)

Data are presented as mean (standard deviation)

A user answers each question with the following score: 7: Strongly agree; 6: Agree; 5: Somewhat agree; 4: Unsure; 3: Somewhat disagree; 2: Disagree; 1: Strongly disagree
